# The signaling pathways of selected traditional Chinese medicine prescriptions and their metabolites in the treatment of diabetic cardiomyopathy: a review

**DOI:** 10.3389/fphar.2024.1416403

**Published:** 2024-07-03

**Authors:** Wencan Li, Xiang Liu, Zheng Liu, Qichang Xing, Renzhu Liu, Qinxuan Wu, Yixiang Hu, Jiani Zhang

**Affiliations:** ^1^ Department of Clinical Pharmacy, Xiangtan Central Hospital, Xiangtan, Hunan, China; ^2^ Hunan Provincial Key Laboratory of the Research and Development of Novel Pharmaceutical Preparations, The “Double-First Class” Application Characteristic Discipline of Hunan Province (Pharmaceutical Science), Changsha Medical University, Changsha, Hunan, China; ^3^ Department of Pharmacy, Xiangtan Central Hospital, Xiangtan, Hunan, China

**Keywords:** diabetic cardiomyopathy, traditional Chinese medicine, signaling pathway, mechanism, review

## Abstract

Diabetic cardiomyopathy (DCM) is a myocardial-specific microvascular disease caused by diabetes that affects the structure and function of the heart and is considered to be the leading cause of morbidity and death in patients with diabetes. Currently, there is no specific treatment or preventive drug for DCM, and there is an urgent need to develop new drugs to treat DCM. Traditional Chinese medicine (TCM) has rich experience in the treatment of DCM, and its characteristics of multi-target, multi-pathway, multi-component, and few side effects can effectively deal with the complexity and long-term nature of DCM. Growing evidence suggests that myocardial fibrosis, inflammation, oxidative stress, apoptosis, cardiac hypertrophy, and advanced glycation end product deposition were the main pathologic mechanisms of DCM. According to the pathological mechanism of DCM, this study revealed the potential of metabolites and prescriptions in TCM against DCM from the perspective of signaling pathways. The results showed that TGF-β/Smad, NF-κB, PI3K/AKT, Nrf2, AMPK, NLRP3, and Wnt/β-catenin signaling pathways were the key signaling pathways for TCM treatment of DCM. The aim of this study was to summarize and update the signaling pathways for TCM treatment of DCM, to screen potential targets for drug candidates against DCM, and to provide new ideas and more experimental evidence for the clinical use of TCM treatment of DCM.

## 1 Introduction

The incidence of diabetes has increased exponentially over the past few decades as living standards have improved, and the number of people worldwide with the disease is reported to be more than 700 million by 2045. The main feature of diabetes is persistent hyperglycemia, which over time will cause endocrine and metabolic disorders in patients with diabetes and lead to complications of diabetes, which is the final outcome of diabetes and the main cause of great pain and economic loss to patients, and has become one of the most serious global public health problems (Li et al., 2022; Huang et al., 2023; Zhou et al., 2023). Diabetic cardiomyopathy (DCM) refers to a complication caused by diabetes, which cannot be explained by hypertensive heart disease, coronary artery disease, valvular heart disease, and other heart diseases, and is characterized by myocardial-specific microvascular disease. It is the leading cause of morbidity and mortality in people with diabetes (Tan et al., 2020; Zhao et al., 2022; Yan et al., 2023).

**FIGURE 1:**
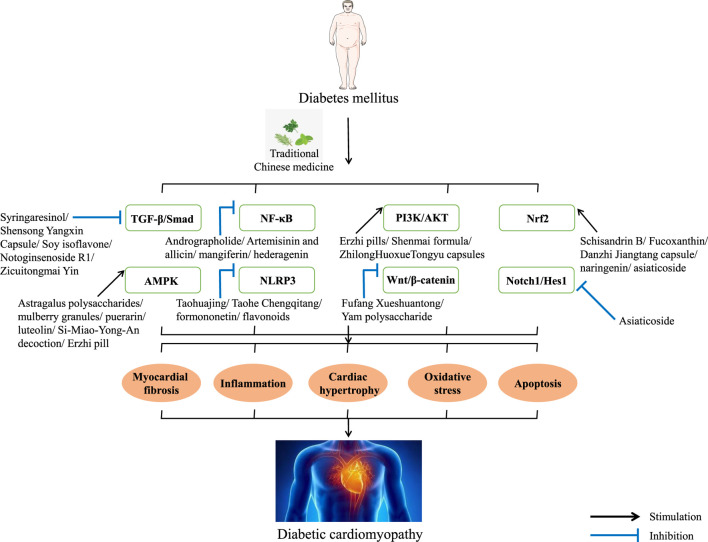
Potential signaling pathways of TCM against DCM.

Clinically, DCM can be divided into two stages according to its typical features. The early stage is characterized by diastolic damage and left ventricular hypertrophy without vascular defects, and the late stage is mainly characterized by systolic dysfunction and myocardial fibrosis (Wang et al., 2022; Zhou et al., 2023). Although the pathogenesis of DCM is complex, after long-term exploration, scholars generally believe that the occurrence and development of DCM is mainly related to glucose and lipid metabolism disorders, microvascular endothelial dysfunction, apoptosis, advanced glycation end product (AGE) deposition, myocardial inflammation, and oxidative stress. Under the influence of the crosstalk of the above factors, it will lead to increased myocardial oxygen consumption, myocardial fibrosis, increased metabolic pressure, significantly reduced cardiac efficiency and function, and ultimately DCM (Huo et al., 2021; Wang et al., 2022; Wu et al., 2023; Cai et al., 2023; Zhou et al., 2023). Currently, there is no specific treatment or preventative drug for DCM. Therefore, it is urgent to explore drugs that are new and have fewer side effects or complementary and alternative therapies for DCM treatment (Tan et al., 2020; Nakamura et al., 2022; Zhou et al., 2023). Traditional Chinese medicine (TCM) has its own unique diagnosis and treatment system, which has been used for the prevention and treatment of diseases for more than 2,000 years in Chinese history. TCM is characterized by multiple targets, multiple pathways, fewer side effects, and easy access. It is considered to be a novel therapeutic agent for many diseases, especially for chronic metabolic diseases such as DCM. In order to better understand the efficacy of TCM, a large number of experimental studies have been carried out, trying to uncover the veil from the molecular mechanism (Zheng et al., 2018; Yang et al., 2021; Li X. H. et al., 2022a). Currently, there is a lack of review of TCM against DCM signaling pathways. This article summarizes the related signaling pathways of TCM against DCM in recent years in order to provide ideas for the development of new drugs against DCM (Figure 1).

## 2 Signaling pathways in TCM intervening the pathological progression of DCM

### 2.1 TGF-β/Smad signaling pathway

Excessive extracellular matrix deposition in the diabetic heart is associated with overexpression of transforming growth factor-β1 (TGF-β1) and connective tissue growth factors, which can cause cardiac fibrosis and damage the structure and function of the heart. When the body is exposed to persistently hyperglycemia levels, endothelial cells take on the characteristics of fibroblasts, leading to diabetic heart fibrosis. TGF-β is one of the key factors in the emergence of DCM that is expressed in all human cells. TGF-β is mainly divided into TGF-β1, TGF-β2, and TGF-β3 forms, which play an important role in myocardial fibrosis, cardiac repair, and cardiac remodeling, especially myocardial fibrosis. TGF-β also has three receptors, namely, TGFBR1, TGFBR2, and TGFBR3. When one of the TGF-β ligands binds to TGFBR2, it will produce typical TGF-β signal transduction, and then, TGFBR2 will recruit and phosphorylate TGFBR1. Phosphorylated TGFBR1 then phosphorylates downstream Smad2 and Smad3 and recruits Smad4 and translocates to the nucleus to bind to DNA and promote TGF-β transcription. Among them, TGF-β1 has a strong anti-inflammatory function and is involved in almost all early tissue damage and is a key cytokine. In addition, Smad3 is pathogenic in cardiovascular diseases, and its overexpression could lead to myocardial inflammation, myocardial fibrosis, and insulin synthesis and secretion disorders, which is a key mediator in the pathogenesis of DCM. Smad7 has a myocardial protective effect, and its overexpression could reverse Smad3-mediated myocardial fibrosis and nuclear factor kappa B (NF-κB)-driven inflammation (Frangogiannis, 2022; Ghafouri-Fard et al., 2023; Qin et al., 2023; Seksaria et al., 2023; Yang et al., 2023).

Syringaresinol (Syr) is a polyphenolic metabolite widely found in various TCM formulations, and many pieces of evidence have shown that Syr has anti-inflammatory, antioxidant, and antitumor activities (Li et al., 2023; Wang et al., 2023; Wang et al., 2023). Li et al. (2020) found that Syr significantly reversed the decrease of ejection fraction (EF), fractional shortening (FS), and cardiac output (CO) in streptozotocin (STZ)-induced diabetic mice and reversed the increase in inflammatory factors tumor necrosis factor-α (TNF-α) and interleukin (IL-6) and the increase in IL-1β and MCP-1 in cardiac tissues of diabetic mice, which indicate that Syr could alleviate cardiac damage and inflammatory response in diabetic mice. Masson trichrome staining and Sirius Red staining showed that Syr decreased interstitial collagen accumulation and effectively reversed myocardial fibrosis in diabetic mice, and Syr significantly decreased the high expression of TGF-β, fibronectin, α-SMA, and p-Smad2/3 in cardiac tissues of diabetic mice. In addition, Syr reversed the increase in ROS and the decrease in SOD in the myocardium of diabetic mice; upregulated the expression of Nrf2, NQO-1, and HO-1; and downregulated the expression of Keap1. In further studies, Li et al. established high-glucose (HG)-treated neonatal rat ventricular myocytes (NRVMs) and verified the abovementioned effects of Syr, and the results showed consistency. These results suggested the potential of Syr to attenuate inflammatory response, myocardial fibrosis, and oxidative stress for the treatment of DCM, and its mechanism of action is related to TGF-β/Smad2/3 and Keap1/Nrf2 signaling pathways.

*Panax notoginseng (Burk.)* F.H. Chen, known as “aspirin” in TCM, has the efficacy of reducing swelling, relieving pain, clearing blood stasis, and stopping bleeding, and has a landmark position in TCM. Notoginsenoside R1 (NGR1) is one of the main bioactive metabolites of *P. notoginseng (Burk.)* F.H. Chen. It has a wide range of protective and therapeutic effects in anti-diabetes, anticancer, and vascular protection, especially cardiovascular and cerebrovascular protection (Liu et al., 2020; Xie and Wang, 2023; Zhu and Wan, 2023). Zhang et al. (2018) found that NGR1 significantly reduced the apoptosis and mitochondrial damage of AGE-induced H9c2 cells *in vitro*; downregulated ROS, Smad2/3, p-Smad2, and collagen I; and promoted the expression of estrogen receptor (ER)-α and Smurf2. *In vivo*, NGR1 decreased LVIDd and LV mass and increased LVVd, LVVs, EF, and FS in diabetic mice. In addition, NGR1 inhibited the increase of LDH, AST, and CK-MB in the serum of diabetic mice. In a further mechanism study, NGR1 upregulated the expression levels of Nrf2, γ-GCS, NQO-1, HO-1, p-AKT, p-GSK-3β, and SnoN and downregulated the expression levels of TGF-β, collagen I, and Smad2/3 in cardiac tissues of diabetic mice. Furthermore, NGR1 promoted the expression levels of ERα and SMurf2 in cardiac tissues of diabetic mice, which was consistent with the results of *in vitro* experiments. These lines of evidence suggest that NGR1 may improve DCM through TGF-β/Smad2/3 and AKT/Nrf2 signaling pathways. Shensong Yangxin capsule (SSYX) is a TCM prescription widely used to treat arrhythmia and has been used clinically for many years (Cao et al., 2021; Ma et al., 2023). Shen et al. (2014) found that SSYX significantly reduced the heart weight/body weight ratio of fat emulsion + STZ-induced diabetic rats and improved the degree of cardiac dysfunction, myocardial fibrosis, and collagen deposition. In addition, it was found in the mechanism study that SSYX downregulated the expression levels of TGF-β1, p-Smad2/3, col-1, col-3, MMP-2, and MMP-9 and upregulated the expression level of Smad7 in myocardial tissues of diabetic rats.

Moreover, Soy isoflavone (Huang et al., 2023), Zicui Tongmai Yin (Wu et al., 2021), ginkgolide B (Gao and Hou, 2022), and echinacoside (Liao et al., 2022) have shown significant effects in alleviating myocardial fibrosis in diabetes by a mechanism related to the inhibition of the TGF-β/Smad signaling pathway.

### 2.2 NF-κB signaling pathway

The nuclear factor kappa B (NF-κB) family consists of five members: p65 (RelA), RelB, c-Rel, p50/p105 (NF-κB1), and p52/p100 (NF-κB2). They are involved in a variety of physiological and pathological processes, including cellular immunity, proliferation, inflammation, and oxidative stress. In DCM, persistently high levels of glucose and LDL/VLDL lipoproteins stimulate NF-κB activation in the myocardium. In addition, they can cause circulating and local cells to release vasoactive polypeptides, TGF-β growth factor, and connective tissue growth factor. These molecules stimulate NF-κB activity either directly or through cytokine-mediated expression. There are two major NF-κB activation pathways in the above process, namely, the canonical and non-canonical signaling pathways. The canonical signaling pathway is mainly induced by pro-inflammatory factor tumor necrosis factor-α (TNF-α), interleukin-1 (IL-1), and lipopolysaccharide (LPS). When cells are stimulated, the IκB kinase (IKK) complex is activated, and signal transduction leads to IκB protein phosphorylation and ubiquitination. IκB is then degraded, releasing the NF-κB dimer from the cytoplasm into the nucleus, where it binds to DNA and promotes its transcription. Among them, IκBα can not only bind to the most common NF-κB heterodimer P65:P50 but also the strongest negative feedback factor in NF-κB activation, ensuring the rapid initiation and termination of the entire process (Lorenzo et al., 2011; Li L. J. et al., 2023; Cheng et al., 2023; Oh et al., 2023).

*Andrographis paniculata* Nees is distributed all over the world and is widely used, and andrographolide (AG) is a diterpene lactone metabolite, which is the main active metabolite of *A. paniculata* Nees and can be used to treat hyperlipidemia, obesity, diabetes, and cardiovascular diseases (Li X. H. et al., 2022b; Gou et al., 2023). Liang et al. (2018) found that diabetes could lead to decreased LVEF, FS, E/A, and insulin levels and increased blood glucose in mice, while AG could reverse the above phenomenon. According to the results of Masson’s trichrome and Sirius Red staining, diabetic mice showed increased collagen deposition in the myocardial interstitial area, and collagen I, collagen indigenous, TGF-β1, fibronectin (FN), ANP, and BNP were decreased; the above phenomena could be significantly improved after AG intervention. In a further mechanism study, the expression levels of COX-2, p-IκBα, p65-NF-κB, intercellular cell adhesion molecule-1 (ICAM-1), vascular cell adhesion molecule-1 (VCAM-1), TNF-α, IL-1β, and IL-6 in myocardial tissues of diabetic mice were increased, while AG could reduce their expression levels. In addition, AG also reduced the expression of MDA, 4-HNE, 3-NT, Nox-2, and Nox-4 and increased the expression of SOD, Nrf2, and HO-1 in myocardial tissues of diabetic mice. In the HG-induced H9c2 cardiomyoblast model, AG reduced the expression of p-IκBα, nucleus-p65-NF-κB, Nox-2, and Nox-4 and promoted the translocation of Nrf2 into the nucleus. These results suggested that AG has the potential to treat DCM by reducing inflammation and oxidative stress, and its mechanism may be related to NF-κB and NOXs/Nrf2 signaling pathways. Artemisinin and allicin (AA) have anticancer, antioxidant, and anti-inflammatory biological activities (Zhou et al., 2022; Jin et al., 2023) and have been proved to be used in clinical anti-myocardial fibrosis (Kong et al., 2023a). Kong et al. (2023a) found that the cardiac function of STZ-induced diabetic rats was damaged, and AA could improve these damages. In addition, the myocardial cells of diabetic rats showed pathological changes, such as increased collagen fibers, a large number of fibrous scars, and increased myocardial cells, and these changes were significantly reduced after AA intervention. In the mechanism study, AA significantly reduced the expression of NF-κB p65 and p-NF-κB p65 in the myocardial tissues of diabetic rats, indicating that the effect of AA on improving myocardial injury and myocardial fibrosis in diabetic rats may be related to the inhibition of the NF-κB signaling pathway. Furthermore, mangiferin (Hou et al., 2016) and hederagenin (Li et al., 2019) play a beneficial role in DCM by inhibiting the NF-κB signaling pathway to reduce the inflammatory response.

### 2.3 PI3K/AKT signaling pathway

Phosphoinositol-3 kinase (PI3K) is an intracellular lipid kinase, which can be divided into three types according to its molecular structure and substrate selectivity. Class I PI3K is the focus of current research. It is a heterodimer composed of a regulatory domain (p85) and a catalytic domain (p110) that phosphorylate the 3′-hydroxyl group of phosphatidylinositol and phosphoinositides. The PI3K-mediated imbalance of fatty acid metabolism and glucose homeostasis in diabetic hearts has been reported to lead to pathological features of DCM, such as decreased cardiac efficiency, pathological myocardial hypertrophy, fibrosis, and apoptosis. Dysregulation of PI3K-induced metabolic imbalances leads to the development of DCM. AKT is also known as protein kinase B (PKB), a serine/threonine kinase widely expressed in human tissues, divided into AKT1 (PKB α), AKT2 (PKB β), and AKT3 (PKB γ), the first two of which are commonly expressed in the heart, brain, and lungs. Overexpression of AKT3 in the heart can induce myocardial hypertrophy. AKT1 and AKT3 function similarly, and it has also been shown that overexpression of AKT1 induces myocardial hypertrophy, but loss of AKT1 leads to resistance to exercise-induced cardiac growth. AKT2 is a regulator of cardiomyocyte metabolism and cardiomyocyte death in DCM. AKT is the main downstream effector molecule of PI3K, which plays a key role in DCM by regulating cell size, survival, apoptosis, angiogenesis, and inflammatory response (Li et al., 2017; Qin et al., 2021; Ghafouri-Fard et al., 2022).

Erzhi pill (EZP) is a TCM prescription composed of the *Ligustrum lucidum* W. T. Aiton and *Eclipta prostrata* (L.) L. in equal proportions. It has the effects of nourishing yin and tonifying the liver and kidney and is commonly used in clinical treatment of diabetic nephropathy and osteoporosis (Li et al., 2020; Peng et al., 2022; Hong et al., 2024). Peng et al. (2022) reported the intervention effect of EZP on diabetic rats induced by high-fat diet combined with STZ. EZP increased the level of high-density lipoprotein (HDL) and decreased the levels of low-density lipoprotein (LDL), triglycerides (TG), total cholesterol (TC), and fasting blood glucose (FBG) in the serum of diabetic rats. In addition, EZP could reverse the decrease in superoxide dismutase (SOD), catalase (CAT), and glutathione peroxidase (GPx) and the increase in caspase-3, caspase-8, caspase-9, reactive oxygen species (ROS), malondialdehyde (MDA), B-cell lymphoma (Bcl)-2, and Bcl-2-associated X protein (Bax) in the serum of diabetic rats. In the study of the mechanism of action, EZP upregulated the expression levels of p-PI3K, p-AKT, p-AMPK, and p-FOXO3a in the heart tissues of diabetic rats. These results suggested that EZP could reduce DCM by inhibiting oxidative stress and apoptosis, and its mechanism is related to PI3K/AKT/FOXO3a and AMPK signaling pathways. The Zhilong Huoxue Tongyu capsule (ZHTC) is a TCM prescription with the effects of tonifying qi, dredging collaterals, promoting blood circulation, and removing blood stasis, which is commonly used in the treatment of ischemic stroke (Wang et al., 2022). Zeng et al. (2023) found that ZHTC could reduce serum GHb, TC, TG, and LDL-C and increase HDL-C in STZ-induced diabetic rats. In addition, the results of HE staining, Masson staining, and TUNEL staining suggested that ZHTC effectively reduced myocardial fibrosis, inflammatory cell infiltration, and apoptosis in diabetic rats. In terms of mechanism, ZHTC upregulated the expression levels of p-PI3K, p-AKT1, and p-FOXO3a in the heart tissues of diabetic rats. These beneficial effects of ZHTC on DCM are related to the regulation of the PI3K/AKT1/FOXO3a signaling pathway.

Moreover, TCM prescriptions Shengjie Tongyu decoction (Wang et al., 2023), Zicui Tongmai decoction (Wu et al., 2023), and Shenmai formula (Li et al., 2023) have shown significant effects in alleviating DCM by a mechanism related to the regulation of the PI3K/AKT signaling pathway.

### 2.4 Nrf2 signaling pathway

Nuclear factor erythroid 2-related factor 2 (Nrf2), a member of the Cap’n’collar (CNC)-BZIP transcription factor family, is a key transcription factor affecting cellular oxidative stress response. Under normal circumstances, Kelch-like ECH-associated protein 1 (Keap1) is a negative regulator of Nrf2, which can bind to Nrf2 in the cytoplasm and form a complex with cullin3, and mediates the degradation of Nrf2 ubiquitination. Under oxidative stress, with the increase in ROS expression, Nrf2 is dissociated from the complex and translocated into the nucleus to bind to the promoter antioxidant response element (ARE) sequence, promoting the expression of Nrf2-related antioxidant enzymes, such as SOD, CAT, heme oxygenase-1 (HO-1), and GPx (Zhang et al., 2021; Li et al., 2023; Ghareghomi et al., 2023). Related studies have shown that Nrf2 is a key regulator of cardiac resistance to ROS in both normal and diabetic hearts. Primary adult cardiomyocytes from mice with deletion of the Nrf2 gene showed isoproterenol-stimulated loss of contraction. In addition, cardiac Nrf2 expression was significantly downregulated in diabetic animals and patients. Clearly, the downregulation of Nrf2 is one of the important reasons for the occurrence of DCM (Chen et al., 2014).

Schisandrin B (SchB) is the main active metabolite of the fruit of *Schisandra chinensis*. It has antioxidant biological activity and exhibits protective effects in many tissues in the body, including the heart (Luo et al., 2022; Yang et al., 2022). Yang et al. (2022) reported the intervention effect of SchB on myocardial injury in STZ-induced diabetic mice. SchB could improve heart weight and the cardiac index; reduce FBG, LVEDD, LVESD, LDH, CK-MB, and cTnI; and increase LVEF and LVFS in the serum of diabetic mice. In addition, HE, Masson, and TUNEL staining results showed that SchB reduced the cardiomyocyte apoptosis rate and collagen volume fraction (CVF). SchB reduced MDA, ROS, and Fe^2+^ and increased SOD and GSH-Px in myocardial tissues of diabetic mice. In terms of mechanism research, SchB upregulated the protein and mRNA expression levels of Nrf2, HO-1, and GPX4 in myocardial tissues of diabetic mice. It is suggested that SchB could improve DCM by exerting antioxidation, anti-fibrosis, and anti-apoptosis effects, and its molecular mechanism may be related to the inhibition of ferroptosis mediated by Nrf2/HO-1/GPX4 signaling pathway activation. Fucoxanthin (FX) is a carotenoid derived from natural marine organisms with antioxidant, anti-diabetic, and anticancer activities (Zheng et al., 2022). Zheng et al. (2022) found that myocardial cells in STZ-induced diabetic rats were hypertrophic and disordered, while FX could improve the above phenomenon. In addition, FX could reduce the surface area of HG-induced H9c2 cells and downregulate the mRNA levels of cell hypertrophy factors ANP, BNP, and β-MHC. These results suggested the ability of FX to reduce cardiomyocyte hypertrophy. In further mechanistic studies, FX reduced the expression levels of ROS, FN, TGF-β1, and Keap1 and upregulated the expression levels of SOD1, nucleus-Nrf2, Nrf2, and HO-1 in heart tissues of diabetic rats. The results suggested that FX has a protective effect on DCM, and its mechanism is related to the Nrf2/Keap1 signaling pathway.

Moreover, naringenin (Li and Lai, 2022), saponins of *Aralia taibaiensis* (Duan et al., 2015), Danzhi Jiangtang capsule (DJC) (Wang et al., 2023), and asiaticoside (Jin et al., 2020) have shown significant effects in alleviating DCM by a mechanism related to the regulation of the Nrf2 signaling pathway.

### 2.5 AMPK signaling pathway

Adenosine monophosphate-activated protein kinase (AMPK) is widely present in eukaryotes and various organs, including three unique subunits of α, β, and γ. AMPK is considered an energy sensor that regulates cellular metabolism, and the regulation of AMPK signaling is crucial for the regulation of intracellular dynamic balance, which is also known as the “total metabolic switch” of the human body. In general, AMPK signaling is activated under low-energy stress conditions. Calcium/calmodulin-dependent protein kinase β (CaMKKβ), TGFβ-activated kinase 1, and liver kinase B1 (LKB1), the upstream mediator of AMPK, can induce T172 phosphorylation in AMPK signaling stimulation. Metabolic stress can trigger AMPK signal transduction by regulating AMP and ATP levels. In this process, increased AMP and adenosine diphosphate levels bind to the γ-subunit site and induce T172 phosphorylation, thereby causing AMPK activation. Moreover, the activation of AMPK can be independent of the regulation of AMP levels. Calcium accumulation in cells causes T172 phosphorylation, resulting in CaMKKβ-dependent upregulation of AMPK, in response to increased intracellular calcium and DNA damage caused by glucose starvation. In addition, AMPK can improve DCM by regulating glycolipid metabolism, regulating protein synthesis and degradation, affecting the transcription of proteins related to gluconeogenesis, lipogenesis, and mitochondrial biogenesis, and regulating vascular endothelial dysfunction. Therefore, AMPK is a potential therapeutic target for DCM (Nellaiappan et al., 2019; Peng et al., 2022; Entezari et al., 2022; Liu et al., 2023).

Astragalus polysaccharide (AP) is a key active ingredient extracted from *Astragalus membranaceus*. Pharmacological studies have shown that AP has anti-inflammatory, antitumor, antioxidation, and anti-fibrosis effects, which is helpful in the management of blood glucose and blood lipids (Zhang and Feng, 2022). Ye et al. (2022) found that LVEDD and LVESD were significantly increased and LVEF and LVFS were significantly decreased in diabetic rats induced by high-sugar and high-fat diets combined with STZ, and these phenomena were significantly reversed after AP intervention. The results of HE staining and Masson staining showed that the myocardial cells of diabetic rats were hypertrophic and disordered, collagen fibers were proliferated, interstitial fibrosis was obvious, and a large number of inflammatory cells were infiltrated. The above conditions were improved after AP intervention. In the mechanism study, AP reduced mTOR and p-mTOR and increased AMPK, p-AMPK protein, and mRNA expression levels in myocardial tissues of diabetic rats. These results indicated that AP could exert a protective effect on DCM by regulating the AMPK/mTOR signaling pathway.

The mulberry granule (MLD), derived from *Morus alba* L., is a TCM prescription that can be used to treat diabetes (Liu et al., 2017). Liu et al. (2017) reported the intervention effect of MLD on STZ-induced diabetic mice. MLD reduced FBG and fasting blood insulin and blood lipids and increased GSH, SOD, CAT, and GR in cardiac tissues of diabetic mice. In addition, MLD could also improve cardiac function and reduce the area of myocardial infarction in diabetic mice. In further mechanism studies, MLD upregulated the expression levels of p-AMPK and Nrf2 cardiac tissues of diabetic mice. The results suggested that MLD may have a protective effect on DCM, and its mechanism may be related to the AMPK/Nrf2 signaling pathway. *Pueraria lobata* (Willd.) Ohwi, as a common TCM, has been made into many kinds of anti-diabetic foods. Puerarin is the main active metabolite of *P. lobata* (Willd.) Ohwi, which has good anti-inflammatory and antioxidant effects (Li et al., 2023). Kong et al. (2023b) found that puerarin significantly reduced the myocardial cell morphology of STZ-induced diabetic rats with irregular and disordered arrangement and a small amount of myocardial cell degeneration and necrosis. The above phenomenon was improved after puerarin intervention. The results of Masson and TUNEL staining showed that puerarin also had a beneficial effect on myocardial fibrosis and apoptosis in diabetic rats. In addition, diabetes resulted in a significant increase in LVESD and LVEDD and a significant decrease in EF and FS in the model rats, which could be reversed by puerarin. Puerarin reduced the levels of FBG, HbA1c, CK-MB, and cTnI in the serum of diabetic rats and upregulated the expression levels of p-AMPK, nucleus-β-catenin, and p-GSK-3β in myocardial tissues. It is suggested that puerarin could improve diabetic myocardial injury by regulating the AMPK/GSK-3β/β-catenin signaling pathway, thus playing a role in protecting DCM. Similarly, Xu et al. (2022) reported that the improvement of puerarin in HG-induced H9c2 cardiomyocyte hypertrophy may be related to the AMPK/AKT/GSK-3β signaling pathway, indicating the importance of AMPK-related signaling pathways in the development of DCM and the great potential of puerarin in the treatment of DCM.

Moreover, luteolin (Huang et al., 2022), Si-Miao-Yong-An decoction (Li et al., 2020), and Erzhi pill (Peng et al., 2022) have shown significant effects in alleviating DCM by a mechanism related to the regulation of the AMPK signaling pathway.

### 2.6 NLRP3 signaling pathway

Nucleotide-binding oligomerization domain-like receptor protein 3 (NLRP3) inflammasome is closely related to metabolic disorders and cell death and is one of the important causes of DCM. One of the important signals during NLRP3 initiation and activation is diabetes-induced hyperglycemia and hyperlipidemia, which can promote ROS overexpression to activate the NF-κB signaling pathway and thus the transcription of NLRP3, pro-IL-1β, and pro-IL-18. Thioredoxin-interacting/inhibiting protein (TXNIP) was another signal during NLRP3 activation that directly combined with NLRP3 to modulate its oligopolization. Hyperglycemia leads to ROS upregulation of TXNIP and thus activation. In addition, hyperglycemia and hyperlipidemia directly exacerbate mitochondrial oxidative stress and proinflammatory cytokine production, thus inducing the formation of NLRP3 inflammasome. Nod-like receptors (NLRs) are a family of pattern recognition receptors (PRRs) used to identify the pathogen-associated molecular pattern (PAMP) and damage-related molecular pattern (DAMP). NLR contains four subfamilies, among which NLRP3 is one of the most representative members. NLRP3 inflammasome consists of NLRP3, pro-caspase-1, and apoptosis-associated speck-like protein containing a CARD domain (ASC). NLRP3 inflammasome activation generally includes two processes. First, when PAMP or DAMP is recognized by the corresponding PRRs, it promotes the transcriptional activation of the NLRP3 inflammasome gene. Then, NLRP3 inflammasome is assembled, caspase-1 is activated, pro-IL-1β and pro-IL-18 shear processing is conducted, and finally, mature forms of IL-1β and IL-18 are produced and secreted extracellularly to trigger the inflammatory response (Luo et al., 2014; Sun and Ding, 2021; Zheng et al., 2022; Jiang et al., 2024).

Taohuajing (THJ) is a TCM prescription consisting of Semen Persicae, *Polygonatum sibiricum*, and *Carthami flos*, which has a good clinical effect in the treatment of DCM (Yao et al., 2021). Yao et al. (2021) observed the intervention effect of THJ on DCM by establishing a high-fat diet combined with an STZ-induced diabetic mouse model. THJ reduced the blood glucose and blood lipid of DCM model mice and improved their insulin sensitivity in a dose-dependent manner. In the study of myocardial function, THJ increased LVEF and LVFS and decreased LVEDV and LVESV in DCM model mice, indicating that THJ could improve myocardial function in DCM model mice. Masson and HE staining showed a significant increase in collagen, myocardial cells and mast cells were destroyed, myofibrils were broken and irregular, and mitochondria were swollen in the heart of DCM model mice, while THJ could reverse these diabetes-induced cardiac function damage. In addition, THJ also reduced the inflammatory factors TNF-α, IL-6, and IL-1β in the serum of DCM model mice, reduced the levels of ROS and MDA, and increased the levels of SOD and GSHPx in heart tissues of DCM model mice, reflecting its ability to inhibit oxidative stress and inflammatory response in DCM model mice. In a further mechanism study, THJ reduced the protein and mRNA expression levels of NLRP3, caspase-1, TXNIP, ASC, and IL-1β in the left ventricular tissues of DCM model mice. In addition, THJ downregulated the expression levels of Ac-SOD2 and AcFOXO3a and upregulated the expression level of SIRT1 in heart tissues of DCM model mice. These experimental results indicated that THJ can protect myocardial injury through antioxidative stress and anti-inflammatory effects, and the molecular mechanism of these effects may be related to the Sirtuin1/NLRP3 signaling pathway. Taohe Chengqitang (TCT), a TCM prescription derived from *Shanghan Zabing Lun*, has the effects of tonifying qi and nourishing yin, promoting blood circulation, and removing blood stasis and could be used clinically to treat diabetic complications (Zhang et al., 2022). Zhang et al. (2022) observed the intervention effect of TCT on diabetic rats. TCT increased EF and FS in STZ-induced diabetic rats and decreased FBG, TC, and TG in the serum of diabetic rats. The inflammatory factors IL-1β and IL-18 in the serum of diabetic rats were significantly increased, and the expression of these inflammatory factors could be significantly reversed after TCT intervention. In the examination of the pathological morphology of myocardial tissues in diabetic rats, it was found that diabetes could lead to hypertrophy of myocardial fibers in rats, disordered arrangement, a small amount of necrotic and apoptotic myocardial fibers, and a small amount of inflammatory cell infiltration, and these symptoms were significantly improved after TCT treatment. In further studies, TCT significantly reduced the expression levels of ASC, caspase-1, NLRP3, and p-NF-κB p65 in myocardial tissues of diabetic rats. It is suggested that TCT could reduce myocardial inflammation and myocardial fibrosis by inhibiting the activation of the NLRP3 inflammasome signaling pathway and improve the cardiac function of DCM rats, thus playing a role in the treatment of DCM.

Moreover, formononetin (Zhang et al., 2023), protocatechualdehyde (Ding et al., 2024), and mulberry leaves flavonoids (Yang and Cao, 2022) have shown significant effects in alleviating DCM by a mechanism related to the inhibition of the NLRP3 signaling pathway.

### 2.7 Wnt/β-catenin signaling pathway

The Wnt/β-catenin signaling pathway is an important regulator of cardiac development and growth. Although its activity is low in the normal heart, it is indispensable for maintaining normal cardiac function, and its long-term high activity can lead to cardiac fibrosis and cardiac hypertrophy. In DCM, hyperglycemia causes an overproduction of ROS through the mitochondrial electron transport chain, causing oxidative stress. Oxidative stress can activate the Wnt/β-catenin signaling pathway and nuclear β-catenin/c-Myc axis and aggravate oxidative stress damage under their cascading influence, thus exacerbating DCM. The Wnt signaling pathway is divided into classical and non-classical pathways; the former mainly controls cell proliferation, while the latter regulates cell polarity and migration, and the two main pathways form a mutual regulatory network. In the canonical Wnt pathway, it activates the Wnt/β-catenin signaling pathway by regulating the downstream key transcription factor β-catenin, and then, the Wnt ligand protein Wnt3α binds to frizzled (Fzd), low-density lipoprotein receptor-related protein receptors 5/6 (LRP5/6) to activate Casein kinase 1 and promote the release of GSK3β-related regulatory proteins. PDZ and DvL are recruited to form a protein complex, which inhibits the activation of GSK3β, thereby inhibiting the degradation of β-catenin. β-catenin can stabilize aggregation and transfer to the nucleus to interact with the T-cell factor/lymphoid enhancer factor, initiating Wnt/β-catenin downstream target gene and protein transcription (Liu et al., 2022; Ma et al., 2023; Ni et al., 2023).

FXST is a TCM prescription composed of *P. notoginseng* (Burk.) F. H. Chen, *Salvia miltiorrhiza* Bunge, *Astragalus membranaceus (Fisch.)* Bunge, and *Scrophularia ningpoensis Hemsl*. It has the function of promoting blood circulation, removing blood stasis, tonifying qi, and nourishing yin and could be used clinically for the treatment of stable angina pectoris and cerebrovascular diseases (Peng et al., 2022; Wang et al., 2022). Peng et al. (2022) observed the intervention effect of FXST on DCM by establishing a STZ-induced diabetic rat model. FXST reduced LVEDP and significantly increased the heart rate, CI, LVSP, dp/dt min, EF, and FS in diabetic rats. Diabetic rats showed myocardial cell disorder, inflammatory cell infiltration, uneven staining of the nucleus and cytoplasm, and a high deposition area of myocardial collagen fibers, which were reversed by FXST intervention. In addition, FXST could reduce the expression levels of collagen I, collagen III, and TGF-β1 in heart tissues of diabetic rats and downregulate the protein expression levels of Wnt2, β-catenin, WISP1, c-Myc, and p-GSK-3β and the mRNA expression levels of β-catenin and c-Myc in myocardial tissues of diabetic rats. These results suggested that FXST could enhance cardiac function and reduce myocardial fibrosis induced by diabetes, thereby improving the role of DCM, which may be related to the downregulation of the Wnt/β-catenin signaling pathway. The work of Song et al. (2023) revealed the intervention of Yam polysaccharide (YP) on HG-induced H9c2 cardiomyocytes. YP decreased the levels of IL-1β, IL-6, ROS, and MDA and increased SOD activity and cell viability in HG-induced H9c2 cardiomyocytes, indicating the anti-inflammatory and antioxidant properties of YP. In terms of mechanism, YP downregulated the expression of Wnt3α and β-catenin and upregulated the expression of GSK3β. These results indicated that YP could improve DCM through anti-inflammatory and antioxidant effects, and its mechanism may be related to the Wnt/β-catenin signaling pathway.

### 2.8 Other signaling pathways

*Centella asiatica* (L.) Urban mainly grows in China and Southeast Asia. It is one of the main TCMs to restore the vitality of nerves and brain cells. Asiaticoside (ASI) is its iconic metabolite, which has anti-inflammatory, antioxidant, and cardioprotective effects (Bandopadhyay et al., 2023). Wei et al. (2022) observed the intervention effect of ASI on myocardial injury in DCM mice. According to the results of Masson staining, it was found that the myocardial fibers of DCM mice were significantly broken and CVF increased, while ASI intervention significantly improved these phenomena. ASI could increase the levels of LVEF, FS, LVEDV, and LVESV and decrease the levels of LVAWd, LDH, and CTGF in DCM mice, which indicated that ASI could reduce cardiac injury and improve cardiac function in DCM mice. In addition, ASI increased the ratio of Beclin1, Atg5, and LC3II/I and downregulated the expression levels of Notch1 and Hes1 in myocardial tissues of DCM mice. These results indicated that ASI can improve DCM by reducing cardiomyocyte autophagy, and its mechanism may be related to the Notch1/Hes1 signaling pathway. Similarly, Yang et al. published their study of ASI against DCM (Yang Z. M. et al., 2021). ASI could improve myocardial structural disorder, myocardial fibrosis, and cardiomyocyte apoptosis in DCM mice by regulating the Notch1/Hes1 signaling pathway, thus protecting DCM. These two studies demonstrated the potential of ASI in the treatment of DCM. DJC is a TCM prescription with the effects of nourishing qi and yin and promoting blood circulation. It has a beneficial effect in the treatment of DCM, cardiovascular diseases, and diabetic nephropathy (Shi et al., 2021; Wang et al., 2023). Shi et al. (2021) found that DJC increased EF and FS levels in DCM rats. DCM rats showed cardiomyocyte dissolution, inflammatory cell infiltration, and an increased apoptosis rate, which could be reversed by DJC intervention. DJC reduced the expression levels of inflammatory factors TNF-α, IL-1β, and IL-6 in the serum of DCM rats and downregulated the expression levels of TLR4, MyD88, and NF-κB p65 in myocardial tissues of DCM rats. In addition, in the *in vitro* study of HG-induced H9c2, DJC could also downregulate the expression levels of TLR4, MyD88, and NF-κB p65, which is consistent with the *in vivo* results. DJC decreased the expression of Bax and caspase-3 and increased the expression of BCL2, reflecting the anti-apoptotic potential of DJC. These results suggested that DJC could treat DCM by inhibiting cardiomyocyte apoptosis *in vitro* and *in vivo*, and its mechanism may be related to the inhibition of the TLR4/MyD88/NF-κB signaling pathway. Furthermore, galangin (Lyu et al., 2022) regulated the IRAK-1/MAPK/NF-κB signaling pathway, gardenoside (Zhang et al., 2021) regulated the VPO1/ERK1/2 signaling pathway, and salvianolic acid B (Zhang and Fu, 2022) regulated the RhoA/ROCK1 signaling pathway to improve DCM.

## 3 Discussion

TCM is a great crystallization of the wisdom generated by ancient Chinese people through clinical practice over a long history and has made an indelible contribution to the prevention and treatment of human diseases. Although TCM has been treating diseases for thousands of years, its focus has always been on the therapeutic effect of diseases, and the mechanism through which TCM exerts its curative effect has not been paid much attention, which is mainly related to the backward cognition and detection methods of people at that time. Modern medicine and TCM have completely different theoretical systems. With the rapid development of modern medicine and the growing popularity of TCM in the world, researchers have begun to explore the mechanisms of TCM and have completed a large number of mechanism studies of TCM against DCM in the past decade.

This review describes the mechanism and potential of TCM against DCM from the perspective of signaling pathways (Table 1). It is noteworthy that TGF-β/Smad, NF-κB, PI3K/AKT, Nrf2, AMPK, NLRP3, and Wnt/β-catenin signaling pathways are prominent in the above studies of TCM against DCM. TCM alleviates DCM mainly by improving pathological processes such as myocardial fibrosis, inflammation, oxidative stress, apoptosis, and cardiac hypertrophy. The above conclusions are also consistent with the current state of research. For example, the TGF-β/Smad signaling pathway is a key signaling pathway associated with cardiovascular disease, especially an important factor in the development of myocardial fibrosis. In this study, Syr, NGR1, SSYX, Soy isoflavone, Zicuitongmai Yin, ginkgolide B, and echinacoside could reduce myocardial fibrosis and improve DCM by regulating the TGF-β/Smad signaling pathway, which proves the great prospect of the TGF-β/Smad signaling pathway in TCM against myocardial fibrosis of DCM. It is well-known that inflammation is one of the main causes of DCM in the initial stage. NF-κB and NLRP3 signaling pathways are two of the most classical inflammatory signaling pathways. In this study, AG, mangiferin, and hederagenin inhibited the NF-κB signaling pathway, and THJ, TCT, and formononetin inhibited the NLRP3 signaling pathway to reduce the inflammatory response of DCM. In addition, oxidative stress is another risk factor for the development of DCM and often occurs in tandem with inflammatory reactions, thus accelerating the development of DCM under the influence of the crosstalk between the two. The Nrf2 signaling pathway is one of the classical signaling pathways for antioxidative stress, and it is also prominent in TCM against oxidative stress of DCM. Syr, NGR1, AG, and SchB have been shown to improve the oxidative stress of DCM by regulating the Nrf2 signaling pathway. Compared to the aforementioned predictive processes, there are no signaling pathways with high relevance in improving TCM regulation of DCM by intervening apoptosis and cardiac hypertrophy, which may be related to the difference in the number of references in the current study of different pathological processes of DCM. Due to the popularity of autophagy research in recent years, there has been a gradual emergence of research on TCM regulation of autophagy to improve DCM, but little research has been done on the signaling pathways involved in autophagy. How TCM interferes with autophagy to improve DCM is a promising research direction and may also be one of the focuses of future research work. In addition, many TCMs improve DCM not only by intervening with one signaling pathway but multiple; for example, AG could reduce the inflammatory response of DCM by inhibiting the activation of the NF-κB signaling pathway and could also improve the oxidative stress of DCM by regulating the Nrf2 signaling pathway; Syr could improve myocardial fibrosis, inflammation, and oxidative stress of DCM by regulating TGF-β/Smad2/3 and Keap1/Nrf2 signaling pathways; NGR1 could improve oxidative stress, apoptosis, and myocardial fibrosis of DCM by regulating TGF-β/Smad2/3 and Akt/Nrf2 signaling pathways; and EZP could improve oxidative stress and apoptosis of DCM by regulating PI3K/Akt/FOXO3a and AMPK signaling pathways. There are many such examples in this study, which reflect the multi-target and multi-pathway characteristics of TCM. Considering the multiple-organ damage caused by hyperglycemia and the complexity and long-term nature of DCM pathogenesis, TCM with multi-target and multi-pathway characteristics and few side effects has broad prospects as a drug candidate for the treatment of DCM. Although TCM has shown great potential in the treatment of DCM, there are still some shortcomings. First, the same TCM in different regions may have different components due to differences in climate, which may lead to differences in the efficacy and toxicity of TCM in application. Second, the complexity of TCM components determines its multi-target and multi-pathway characteristics, which is an advantage of TCM but also a disadvantage, making it more difficult to study the specific mechanism of TCM against DCM. Third, there is a lack of large clinical trials of TCM in the treatment of DCM. Fourth, some TCM prescriptions are hospital preparations whose production process standards are not clear and uniform. Finally, it is worth noting that the scientific quality of the references needed to be assessed, and based on the available information, we defined (A) experiments containing animal and cellular experiments with positive or negative control groups; (B) experiments containing animal and cellular experiments without positive or negative control groups; (C) experiments containing only animal experiments; and (D) experiments containing only cellular experiments. Obviously, the evidence of type A not only obtains relevant results from *in vivo* experiments but can also be further verified by *in vitro* experiments and positive or negative control groups to confirm the reliability of the results, which is a higher level of experimental evidence. The level of the evidence of type B is only second to that of type A, which also belongs to a higher level of experimental evidence. The evidence of type C and type D belongs to a slightly lower level of evidence relative to A and B, especially type D. Due to the singularity of the cell itself and its growth environment, which is far less complex than that of animals, often evidence that appears in cell experiments is not necessarily validated in animal experiments, and thus, type D belongs to a lower level of evidence among these. In short, all the evidence needs to be viewed dialectically. In addition, dose is a very important event in pharmacological studies, and for *in vivo* studies, as a general rule, the dose tested should not exceed 1 g/kg and day. However, a small number of TCM prescriptions included in this review have doses exceeding 1 g/kg and day, and the maximum dose even reaches 4 g/kg and day, which is worth considering. If an excessive dose is required to achieve the relevant pharmacological activity, can it be reasonably assumed that it has no activity at lower doses and, thus, that they are not pharmacologically relevant? Even in excessive doses, the toxicity of TCM should be evaluated. Therefore, this review suggests that future pharmacological studies should be evaluated at more relevant dose levels to make the conclusions more convincing. The relevant basic pharmacological data are shown in Table 2.

**TABLE 1:** *In vivo* and *in vitro* experimental evidence of TCM in the treatment of DCM.

Type of signaling pathways	Signaling pathways	Agents	Plants	Experiment model		Molecular mechanisms	Pathological progressions	References
				*In Vivo*	*In Vitro*			
TGF-β/Smad	TGF-β/Smad2/3 and Keap1/Nrf2	Syringaresinol (Syr)	-	C57BL/6 mice	NRVMs	↓: TGF-β, fibronectin, MCP-1, α-SMA, p-Smad2/3, ROS, and Keap1 ↑: Nrf2, NQO-1, HO-1, SOD, and nucleus-Nrf2	Myocardial fibrosis, inflammation, and oxidative stress	Li et al. (2020a)
	TGF-β/Smad2/3, AKT/Nrf2	Notoginsenoside R1 (NGR1)	*Panax notoginseng (Burk.)* F.H. Chen	db/db mice	H9c2 cardiomyocytes	↓: ROS, TGF-β, Smad2/3, p-Smad2, Collagen I, cleaved caspase-3, cleaved caspase-9, and Bax/Bcl-2 ↑: nucleus-Nrf2, NQO-1, HO-1, γ-GCS, ER-α, Smurf2, Nrf2, p-AKT, p-GSK-3β, and SnoN	Oxidative stress, apoptosis, and myocardial fibrosis	Zhang et al. (2018)
	TGF-β1/Smad	Shensong Yangxin capsule (SSYX)	*Panax ginseng* C.A. Mey., *Salvia miltiorrhiza* Bge., *Nardostachys jatamansi* Dc., *Cornus officinalis* Sieb.et Zucc., *Taxillus chinensis* (DC.) Danser, *Paeonia lactiflora* Pall., *Schisandra sphenanthera* Rehd. et, *Coptis chinensis* Franch., *Ophiopogon japonicas* (Thunb.) Ker-Gawl., *Polypodiodes chinensis*, *Eupolyphaga sinensis* Walker, and *Ziziphus jujuba* Mill. var. spinosa (Bunge) Hu ex H. F. Chou	Wistar rats	-	↓: TGF-β1, p-Smad2/3, col-1, col-3, MMP-2, and MMP-9 ↑: Smad7	Myocardial fibrosis	Shen et al. (2014)
NF-κB	NF-κB, NOXs/Nrf2	Andrographolide (AG)	*Andrographis paniculata* Nees	C57/BL6J mice	H9c2 cardiomyoblasts	↓: Collagen I, collagen Ш, TGF-β1, COX-2, p-IκBα, p65-NF-κB, ICAM-1, VCAM-1, TNF-α, IL-1β, IL-6, MDA, 4-HNE, 3-NT, Nox-2, Nox-4, and nucleus-p65-NF-κB ↑: SOD, Nrf2, HO-1, and nucleus-Nrf2	Inflammation, oxidative stress	Liang et al. (2018)
	TLR4/MyD88/NF-κB	Danzhi Jiangtang capsule (DJC)	*-*	SD rats	H9c2 cells	↓: TLR4, MyD88, NF-κB p65, TNF-α, IL-1β, IL-6, Bax, and caspase-3 ↑: BCL2	Apoptosis	Shi et al. (2021)
PI3K/AKT and AMPK	PI3K/AKT/FOXO3a and AMPK	Erzhi pills (EZP)	*Ligustrum lucidum* W.T. Aiton and *Eclipta prostrata* (L.) L	SD rats	-	↓: ROS, MDA, caspase-3, caspase-8, caspase-9, LDL, TG, TC, and FBG ↑: p-PI3K, p-AKT, p-FOXO3a, p-AMPK, HDL, SOD, CAT, and GPx	Oxidative stress and apoptosis	Peng et al. (2022b)
AMPK	AMPK/GSK-3β/β-catenin and AMPK/AKT/GSK-3β	Puerarin	*Pueraria lobata* (Willd.) Ohwi	SD rats	H9c2 cardiomyocytes	↓: CK-MB, cTnI, β-MHC, and IP3R2 ↑: p-AMPK, p-GSK-3β, nucleus-β-catenin, p-AMPKα, and p-AKT	Myocardial fibrosis, apoptosis, and cardiac hypertrophy	Kong et al. (2023b), Xu et al. (2022)
Nrf2	Nrf2/HO-1/GPX4	Schisandrin B (SchB)	*Schisandra chinensis*	C57BL/6 mice	-	↓: MDA, ROS, and Fe2+ ↑: Nrf2, HO-1, GPX4, SOD, and GSH-Px	Oxidative stress, myocardial fibrosis, and apoptosis	Yang et al. (2022)
	Nrf2/Keap1	Fucoxanthin (FX)	*-*	SD rats	H9c2 cardiomyocytes	↓: TGF-β1, Keap1, ROS, ANP, BNP, β-MHC, and FN ↑: SOD1, nucleus-Nrf2, Nrf2, and HO-1	Myocardial fibrosis and cardiac hypertrophy	Zheng et al. (2022b)
NLRP3	Sirtuin1/NLRP3	Taohuajing (THJ)	Semen Persicae, *Polygonatum sibiricum*, and *Carthami flos*	C57BL/6 mice	-	↓: NLRP3, caspase-1, TXNIP, ASC, Ac-SOD2, AcFOXO3a, ROS, MDA, TNF-α, IL-6, and IL-1β ↑: SIRT1, SOD, and GSHPx	Oxidative stress and inflammation	Yao et al. (2021)
Wnt/β-catenin	Wnt/β-catenin	Fufang Xueshuantong (FXST)	*Panax notoginseng* (Burk.) F. H. Chen, *Salvia miltiorrhiza* Bunge, *Astragalus membranaceus (Fisch.)* Bunge, and *Scrophularia ningpoensis Hemsl*	SD rats	-	↓: Collagen I, collagen III, TGF-β1, Wnt2, β-catenin, WISP1, c-Myc, and p-GSK-3β	Myocardial fibrosis	Peng et al. (2022c)
	Wnt/β-catenin	Yam polysaccharide (YP)	*Dioscorea opposita* Thunb	-	H9c2 cardiomyocytes	↓: IL-1β, IL-6, ROS, MDA, Wnt3α, and β-catenin ↑: GSK-3β and SOD	Oxidative stress and inflammation	Song et al. (2023)

↑, upgrade; ↓, downgrade.

**TABLE 2:** Basic pharmacological data from *in vivo* and *in vitro* experiments on TCM treatment of DCM.

Agent	Type of agents	Experiment model		Dose range tested/administration	Duration	Positive and negative controls	References
		*In Vivo*	*In Vitro*				
Syringaresinol (Syr)	Metabolite	Male C57BL/6 mice, 18–22 g, 6–8 w, STZ-induced model	NRVMs, HG-induced model	*In vivo*: 25 mg/kg (gavage every other day) *In vitro*: 50 and 100 µM	*In vivo*: 8 w	-	Li et al. (2020a)
Notoginsenoside R1 (NGR1)	Metabolite	Female db/db mice, 6–8 w	H9c2 cardiomyocytes, AGE-induced model	*In vivo*: 7.5, 15, and 30 mg/kg (gavage) *In vitro*: 25 µM	*In vivo*: 20 w *In vitro*: 24 h	Positive control: metformin 200 mg/kg (*in vivo*)	Zhang et al. (2018)
Shensong Yangxin capsule (SSYX)	TCM prescription	Male Wistar rats, 180–220 g, fat emulsion + STZ-induced model	-	*In vivo*: 50, 100, and 200 mg/kg	-	-	Shen et al. (2014)
Andrographolide (AG)	Metabolite	C57/BL6J mice, 25–30 g, 8 w, STZ-induced model	H9c2 cardiomyoblasts, HG-induced model	*In vivo*: 1, 10, and 20 mg/kg (gavage) *In vitro*: 0.1, 1, 5, and 10 µM	*In vivo*: 12 w	-	Liang et al. (2018)
Danzhi Jiangtang capsule (DJC)	TCM prescription	Male SD rats, 200 ± 20 g, 8–9 w, high-energy diet + STZ-induced model	H9c2 cells, HG-induced model	*In vivo*: 270, 540, and 1,080 mg/kg (gavage) *In vitro*: 15% DJC-containing serum	*In vivo*: 8 w *In vitro*: 48 h	Positive control: TAK242 1.0 µM (*in vitro*)	Shi et al. (2021)
Erzhi pills (EZP)	TCM prescription	Male SD rats, 190 ± 10 g, 6 w, high-fat diet + STZ-induced model	-	*In vivo*: 1, 2, and 4 g/kg (gavage)	*In vivo*: 8 w	-	Peng et al. (2022b)
Puerarin	Metabolite	Male SD rats, 170 ± 10 g, STZ-induced model	H9c2 cardiomyocytes, HG-induced model	*In vivo*: 20 and 40 mg/kg (intraperitoneal injection) *In vitro*: 5 and 20 µM	*In vivo*: 5 days *In vitro*: 48 h	Negative control: puerarin 40 mg/kg + compound C 1 mg/kg (*in vivo*)	Kong et al. (2023b), Xu et al. (2022)
Schisandrin B (SchB)	Metabolite	Male C57BL/6 mice, 18–20 g, STZ-induced model	-	*In vivo*: 50 and 100 mg/kg (gavage)	*In vivo*: 4 w	Negative control: SchB 100 mg/kg + ML385 30 mg/kg (*in vivo*)	Yang et al. (2022)
Fucoxanthin (FX)	Metabolite	Male SD rats, 200 ± 10 g, STZ-induced model	H9c2 cardiomyocytes, HG-induced	*In vivo*: 200 mg/kg (gavage) *In vitro*: 1 µM	*In vivo*: 12 w *In vitro*: 48 h	Positive control: metformin 230 mg/kg (*in vivo*)	Zheng et al. (2022a)
Taohuajing (THJ)	TCM prescription	C57BL/6 mice, 23–25 g, 8–10 w, high-fat diet + STZ-induced model	-	*In vivo*: 125, 250, and 500 mg/kg (gavage)	*In vivo*: 12 w	-	Yao et al. (2021)
Fufang Xueshuantong (FXST)	TCM prescription	Male SD rats, 180–200 g, STZ-induced model	-	*In vivo*: 1,050 mg/kg (gavage)	*In vivo*: 12 w	-	Peng et al. (2022c)
Yam polysaccharide (YP)	Metabolite	-	H9c2 cardiomyocytes, HG-induced	*In vitro*: 0.2 mg/mL	*In vitro*: 48 h	Negative control: SchB 0.2 mg/mL + LiCl 40 µM (*in vitro*)	Song et al. (2023)

In summary, in the future, more in-depth studies should be conducted to explore the pathogenesis of DCM and screen potential targets for drug candidates against DCM, thus providing new ideas and more experimental evidence for the clinical use of TCM in the treatment of DCM.
